# Dietary gap assessment: an approach for evaluating whether a country’s food supply can support healthy diets at the population level

**DOI:** 10.1017/S1368980017001173

**Published:** 2017-06-21

**Authors:** Edye M Kuyper, Reina Engle-Stone, Joanne E Arsenault, Mary Arimond, Katherine P Adams, Kathryn G Dewey

**Affiliations:** 1 University of California, Davis, College of Agricultural and Environmental Sciences, International Programs Office, Davis, CA, USA; 2 University of California, Davis, Program in International and Community Nutrition, 3253 Meyer Hall, One Shields Avenue, Davis, CA 95616, USA

**Keywords:** Dietary diversity, Food supply, Food balance sheets, Dietary Approaches to Stop Hypertension diet, Agriculture–nutrition

## Abstract

**Objective:**

Dietary diversity, and in particular consumption of nutrient-rich foods including fruits, vegetables, nuts, beans and animal-source foods, is linked to greater nutrient adequacy. We developed a ‘dietary gap assessment’ to evaluate the degree to which a nation’s food supply could support healthy diets at the population level.

**Design/Setting:**

In the absence of global food-based dietary guidelines, we selected the Dietary Approaches to Stop Hypertension (DASH) diet as an example because there is evidence it prevents diet-related chronic disease and supports adequate micronutrient intakes. We used the DASH guidelines to shape a hypothetical ‘healthy’ diet for the test country of Cameroon. Food availability was estimated using FAO Food Balance Sheet data on country-level food supply. For each of the seven food groups in the ‘healthy’ diet, we calculated the difference between the estimated national supply (in kcal, edible portion only) and the target amounts.

**Results:**

In Cameroon, dairy and other animal-source foods were not adequately available to meet healthy diet recommendations: the deficit was −365 kcal (–1527 kJ)/capita per d for dairy products and −185 kcal (–774 kJ)/capita per d for meat, poultry, fish and eggs. Adequacy of fruits and vegetables depended on food group categorization. When tubers and plantains were categorized as vegetables and fruits, respectively, supply nearly met recommendations. Categorizing tubers and plantains as starchy staples resulted in pronounced supply shortfalls: −109 kcal (–457 kJ)/capita per d for fruits and −94 kcal (–393 kJ)/capita per d for vegetables.

**Conclusions:**

The dietary gap assessment illustrates an approach for better understanding how food supply patterns need to change to achieve healthier dietary patterns.

The need to improve diet quality, particularly among populations most vulnerable to malnutrition, currently figures prominently in global conversations^(^ ^1^ ^)^. The ability to access a healthy diet is included in the definition of food security: ‘Food security exists when all people, at all times, have physical and economic access to sufficient, safe and nutritious food that meets their dietary needs and food preferences for an active and healthy life’^(^ ^2^ ^)^. At the country level, population access to a high-quality diet depends on the availability of an adequate supply of diverse nutrient-rich foods^(^ ^3^ ^)^. Availability of such foods is not sufficient to ensure consumption of a healthy diet by everyone in the population, due to variation in food distribution, access and utilization attributable to factors such as geographic location, gender, age or socio-economic status^(^ ^4^ ^)^. Nevertheless, improving the adequacy of a nation’s overall food supply is an essential step in meeting dietary intake goals. The objective of the present paper is to describe the methodology for a ‘dietary gap assessment’ approach to evaluating the degree to which a nation’s current food supply could meet the goal of achieving ‘healthy’ diets. To demonstrate our methodology, we chose one healthy diet pattern^(^ ^5^ ^)^ and assessed food availability to meet this pattern in one country, Cameroon.

Adherence to dietary guidance and recommended consumption patterns is associated with reductions in chronic disease risk and all-cause mortality in adults^(^ ^6^ ^,^ ^7^ ^)^. In low-income settings, dietary diversity has been linked to higher micronutrient density in infant diets and a higher probability of micronutrient adequacy for adult women^(^ ^8^ ^)^. Diets in low-income countries and communities are often dominated by starchy staple foods, with much smaller amounts of nutrient-dense foods such as fruits, vegetables, nuts, beans and animal-source foods^(^ ^9^ ^)^. Compared with most staple foods, these latter foods generally have a higher nutrient density (amount of critical nutrients per unit of energy). Country-level supply of starchy cereal and tuber foods is associated with child stunting^(^ ^10^ ^)^, possibly due to the low micronutrient density of available foods. At the same time, countries undergoing the ‘nutrition transition’ are experiencing greater demand for foods high in fat and sugar, as well as rising rates of obesity and associated chronic diseases^(^ ^11^ ^)^. Cameroon, the ‘test country’ used to demonstrate the approach described here, is among the countries currently experiencing the ‘triple burden’ of undernutrition, micronutrient malnutrition and diet-related chronic disease^(^ ^12^ ^)^. These populations continue to experience undernutrition, including micronutrient deficiencies, often resulting in the coexistence of over- and undernutrition in the same households and even at the level of the individual (e.g. a person who is simultaneously obese, stunted and anaemic)^(^ ^13^ ^,^ ^14^ ^)^. Global energy availability per capita is continuing to increase, and is associated with reduced rates of child stunting and (although non-significant) increased rates of IHD^(^ ^15^ ^)^. Given that no country to date has reversed its obesity epidemic^(^ ^16^ ^)^ and that rates are growing rapidly even in low-income countries^(^ ^17^ ^–^ ^19^ ^)^, addressing obesity-related lifestyle factors, including diet quality, is necessary in all countries.

It has been argued that improvements to diet quality offer a sustainable path to addressing the multiple forms of malnutrition^(^ ^20^ ^)^. Nutrient fortification and supplementation interventions are efficacious in addressing micronutrient malnutrition, but sustained success at scale depends on external funding or adequate consumer demand^(^ ^21^ ^,^ ^22^ ^)^. Additionally, such interventions do little to address non-communicable diseases and may even exacerbate non-communicable diseases in instances where the vehicle for fortification does not support healthy eating patterns (e.g. vitamin A-fortified chocolate wafers). Dietary improvements similarly require consumer demand and access, but can simultaneously address the challenges of nutrition-related chronic disease, micronutrient malnutrition and undernutrition. Dietary change requires contributions from public- and private-sector actors working in health and agriculture^(^ ^3^ ^,^ ^23^ ^)^ and comprehensive policies on health, agriculture and trade that promote healthy diets in the face of globalization^(^ ^24^ ^)^. In this context, better understanding of how the current food supply needs to change may help inform efforts to achieve healthier diets^(^ ^23^ ^,^ ^25^ ^)^.

Although a universal definition of a healthy diet does not exist, various food-based dietary guidelines have been issued that translate recommended nutrient intakes into specific recommendations for foods to consume or avoid for optimal health. Food-based guidelines have been developed for over 100 countries, including numerous upper- and middle-income countries but few lower-income countries^(^ ^26^ ^)^. In developing food-based dietary guidelines, national authorities consider evidence linking diet and health in the context of national public health issues and priorities and cultural dietary patterns. Once national health and nutrition priorities have been identified, locally available, culturally acceptable foods that address nutrient requirements are identified^(^ ^27^ ^)^. While guidelines and particularly specific food items vary across countries, there are common elements, including recommendations to consume a variety of food groups, to increase consumption of fruits and vegetables, and to limit consumption of foods associated with chronic disease risk^(^ ^28^ ^)^. In addition to national food-based dietary guidelines, other approaches have included identification of healthful diet patterns at the regional level (e.g. the Mediterranean diet^(^ ^29^ ^)^, the new Nordic diet^(^ ^30^ ^)^) and for reduction in risk of specific non-communicable diseases (e.g. the Dietary Approaches to Stop Hypertension (DASH) diet^(^ ^31^ ^)^). These diets also include the same elements of diversity, ample fruits and vegetables, and moderation in consumption of certain foods and ingredients^(^ ^32^ ^,^ ^33^ ^)^. The global relevance of these common elements is also affirmed in WHO guidance^(^ ^34^ ^)^. For our analysis, we selected the DASH diet pattern as an example because there is strong evidence (including a randomized trial, albeit in the US context) that it leads to reductions in multiple biomarkers of chronic disease risk factors^(^ ^35^ ^)^ while also meeting nutrient requirements^(^ ^36^ ^)^. We selected Cameroon as the ‘test country’ because we had access to nationally representative dietary data to guide the categorization of foods commonly consumed in the local diet into the food groups set forth in the DASH diet, to account for differences in local food availability and preferences when applying this diet to other contexts.

The method described in the current paper consists of the following steps: (i) use the DASH guidelines, which are expressed in numbers of servings per food group, to construct a reference ‘healthy’ diet expressed as the average amount of energy to be consumed from each of seven food groups, per capita, per day; (ii) calculate the quantity of food within each of the food groups that would be required for all Cameroonians to attain the reference diet; and (iii) compare the results with national-level data on food supply from FAO Food Balance Sheets (FBS). This allows us to estimate the ‘gap’ between the current food supply and that required to promote health. Although other efforts have focused on global availability of selected foods^(^ ^37^ ^)^ and regional^(^ ^38^ ^)^ and national availability of specific nutrients^(^ ^39^ ^,^ ^40^ ^)^, to our knowledge, this is the first attempt to develop an adaptable methodology that could be used, globally, to identify gaps between national food supplies and an evidence-based healthy diet pattern. Because our approach uses FAO FBS data to quantify the food supply, the approach could be extended globally and could incorporate evaluation relative to other reference diet patterns.

## Methods

### Overview

To estimate the dietary gap in Cameroon, we: (i) selected energy intake per food group as the most appropriate metric for comparing the food supply data with the DASH diet recommendations; (ii) used intake data from Cameroon to select foods commonly consumed in Cameroon to be included in each of the DASH food groups; (iii) calculated target energy values for each food group (kcal/capita per d); (iv) used national food supply data to calculate per capita energy supply for each DASH food group; and (v) compared the national food supply with target energy values. Each of these steps is described below.

### Rationale for using the DASH diet

Research preceding the development of the DASH diet assessed the impact of single nutrients and dietary factors on reducing rates of hypertension. The DASH study was initiated to evaluate the efficacy of an overall dietary pattern, rather than single nutrients, for lowering blood pressure^(^ ^31^ ^)^. The DASH diet was designed to help ensure adequate intakes of both macronutrients and micronutrients, as described in greater detail elsewhere^(^ ^5^ ^,^ ^36^ ^)^. It was developed to be consistent with dietary recommendations for the prevention of cancer, osteoporosis and heart disease^(^ ^41^ ^)^, and emphasizes low sodium intake, increased consumption of fruits, vegetables and wholegrain cereals, and balanced intake of lean meats, poultry, fish, eggs and low-fat dairy products.

### Rationale for selecting Cameroon as test country

Like many low- and middle-income countries, Cameroon is experiencing a nutrition transition, with slightly over half the population now residing in urban areas^(^ ^42^ ^)^. The *Global Nutrition Report 2015* cites high rates of stunting in Cameroon among children under the age of 5 years (33 %) and anaemia among women of reproductive age (41·5 %)^(^ ^43^ ^)^, and a nationally representative survey showed that 32 % of women of reproductive age were overweight (BMI≥25 kg/m^2^) and 11 % were obese (BMI≥30 kg/m^2^). Overweight and obesity were particularly evident in the two largest urban areas, where 48 % of women had BMI≥25 kg/m^2^ and 20 % had BMI≥30 kg/m^2^^(^ ^44^ ^)^. Because Cameroon is experiencing the nutrition transition and because data were available from a 2009 national nutrition survey including dietary intake data that enabled us to operationalize the DASH diet, Cameroon was a logical choice for a first test case of the dietary gap approach.

### Selection of energy as the unit of comparison

The DASH dietary guidelines recommend consuming a specific number of servings of food from each of seven food groups. To operationalize the DASH recommendations for our analysis, we chose to convert the recommended number of servings for each food group into total energy (in kilocalories; 1 kcal=4·184 kJ) from each group, rather than amounts (grams) per group, to allow for the most appropriate comparison with the data available from the FBS (described further below). In particular, the FBS data for weight in grams represent the total amount of food available as it enters the household, including any non-edible (waste) portion, whereas the values for energy equivalents (kcal from each food) represent the edible portion only and thus are more appropriate for comparison with a reference diet that is also based on edible portions.

We set the target energy content of the reference diet to 2100 kcal (8786 kJ)/capita per d based on the World Food Programme’s estimated energy requirements for populations^(^ ^45^ ^)^. The World Food Programme’s estimate is based on a generic developing-country profile assuming an adult male weight of 60 kg, an adult female weight of 52 kg and ‘light’ physical activity level. Using the World Food Programme’s age- and sex-specific estimates of per capita energy requirements, we calculated a weighted average of daily energy requirements based on Cameroon’s age and sex profile as well as the proportions of pregnant and lactating women^(^ ^46^ ^)^. The resulting estimated energy requirement (2040 kcal (8535 kJ)/capita per d) was similar to the World Food Programme’s estimate, so for simplicity we based our calculations on a 2100 kcal (8786 kJ) reference diet.

### Classification of local foods into reference diet groups

By presenting a dietary pattern based on food groups, the DASH diet allows flexibility to select food items within each food group that are preferred based on cultural norms, tastes, allergies, etc. However, the diversity of foods within each food group complicates the calculation of a single value for energy content per serving from a food group, which will depend on the individual food items selected.

Because the DASH diet was designed based on foods commonly consumed in the USA, assessing the adequacy of the national food supply in Cameroon to achieve the DASH diet required populating the DASH food groups with foods consumed in Cameroon. This was accomplished by first selecting a ‘short list’ of available and accessible food items in Cameroon to represent each food group (Table 1). These items were identified by examining data from a national survey of dietary intakes of women and young children in Cameroon^(^ ^47^ ^)^. Dietary data were collected using an interactive 24 h recall method, with replicate recalls in a subset of participants. Food items were included in the short list if they were consumed on at least ~5 % of person-days in the dietary database. Spices, alcoholic beverages and pastries (from the Grains group) and items in the ‘Sweets and added sugars’ food group (refined sugar and chocolate products) were excluded.

**Table 1:** Description of the reference dietary pattern based on the Dietary Approaches to Stop Hypertension (DASH) diet and adjusted to include food items that are commonly consumed in Cameroon

			Scenario A, starchy fruits and vegetables are categorized with other starchy staples				Scenario B, starchy fruits and vegetables are categorized with other fruits and vegetables			
DASH food group	Estimated no. of servings/d recommended by DASH for 2000 kcal (8368 kJ) diet*	Example serving sizes	‘Short list’ of food items selected from dietary data set† to represent food group	No. of servings/d, adjusted to 2100 kcal (8786 kJ)/capita per d	Average energy content per serving using Cameroonian food patterns (kcal/serving)	Average energy content of food group per day in Cameroon DASH pattern (kcal/capita per d)‡	‘Short list’ of food items selected from dietary data set† to represent food group	No. of servings/d, adjusted to 2100 kcal (8786 kJ)/capita per d	Average energy content per serving using Cameroonian food patterns (kcal/serving)	Average energy content of food group per day in Cameroon DASH pattern, (kcal/d)‡
Dairy	2·5	1 cup milk or yoghurt	Fresh whole cow’s milk, powdered cow’s milk, whole yoghurt	2·5	162	409	Fresh whole cow’s milk, powdered cow’s milk, whole yoghurt	2·3	162	373
Fats and oils	2·5	1 teaspoon of oil or margarine	Groundnut, palm, cottonseed and palm kernel oil; margarine	2·5	39	98	Groundnut, palm, cottonseed, and palm kernel oil; margarine	2·3	39	89
Fruits	4·5	1 medium fruit; ½ cup fresh fruit	Bananas, oranges, lemons/limes, papayas	4·5	45	203	Bananas, oranges, lemons/limes, papayas, plantains	4·1	56	233
Grains (Starchy staples)	7·0	1 slice bread; ½ cup cooked rice, pasta or cereal	Pasta/macaroni, bread, rice, rice flour, maize, maize flour, millet, millet flour, sorghum, plantains, Irish potatoes, sweet potatoes, cassava, yam, taro, cocoyam	7·1	115	813	Pasta/macaroni, bread, rice, rice flour, maize, maize flour, millet, millet flour, sorghum	6·4	117	752
Meat, poultry, fish and eggs	5·0	1 oz (28·35 g) cooked meat or fish; 1 egg	Fish (ocean and freshwater), crustaceans, beef, sheep meat, goat meat, pork, chicken eggs	5·1	59	300	Fish (ocean and freshwater), crustaceans, beef, sheep meat, goat meat, pork, chicken eggs	4·6	59	273
Nuts, seeds and legumes	0·7	½ cup cooked legumes, 2 tablespoons seeds	Beans, cowpeas, groundnuts, groundnut paste, melon seed	0·7§	163	117	Beans, cowpeas, groundnuts, groundnut paste, melon seed	0·7§	163	107
Vegetables	4·5	½ cup cooked vegetable	Tomatoes, onions, leeks, okra, cassava leaves, string beans, carrots, other vegetables (green leafy vegetables)	4·5	35	160	Tomatoes, onions, leeks, okra, cassava leaves, string beans, carrots, other vegetables (green leafy vegetables), Irish potatoes, sweet potatoes, cassava, yam, taro, cocoyam	4·1	66	272
Total kcal/d						2100				2100

1 kcal=4·184 kJ.

* See FAO^(^ ^53^ ^)^.

† See Engle-Stone *et al*.^(^ ^44^ ^)^.

‡ Some values may differ from the product of the previous two columns due to rounding of the average kcal/serving.

§ Calculated from 5 servings/week. The adjusted (2100 kcal (8786 kJ)) value is slightly lower but rounds to 0·7 servings/d.

Then, we classified each food on the short list into one of the seven DASH groups. For most foods, classification was straightforward (dairy, fats and oils, grains, meat and nuts/seeds/legumes). However, definitions of fruit and vegetable groups historically have varied depending on context and objective^(^ ^48^ ^)^. In the broadest definition, ‘vegetable’ could refer to any edible plant. More commonly, grains and legumes/nuts/seeds are grouped separately. Classifications of roots and tubers such as white potatoes, sweet potatoes and yams have varied depending on cultural context and the purpose of the classification. Some have advocated excluding all roots and tubers from the vegetable category^(^ ^49^ ^)^ and some have also excluded plantains from the fruit category^(^ ^50^ ^,^ ^51^ ^)^. The rationale for these exclusions is that these foods are different from other fruits and vegetables in both nutrient content and culinary use, and typically function as ‘starchy staples’ in the areas where they are consumed. However, in the DASH diet, white potatoes are classified as a vegetable. Because the DASH diet was designed to include only foods commonly consumed in the USA, there is no mention in the original DASH diet description of other roots and tubers commonly consumed in tropical countries nor is there mention of plantains^(^ ^52^ ^)^. We took two approaches to classifying roots, tubers and plantains in order to illustrate the impact of these classifications on assessment of the adequacy of the national supply of fruits and vegetables. In the first scenario, we included plantains and all roots and tubers (white potato, sweet potato, yam, taro and cocoyam) in the ‘Starchy staples’ category (Table 1, Scenario A). In the second scenario, plantains were classified as fruits, and all roots and tubers were classified as vegetables (Table 1, Scenario B).

We also had to decide how to treat dairy products, as the DASH diet recommends consumption of low-fat dairy products but these products are scarce in Cameroon. In the interest of constructing a food list reflective of available products, we retained the full-fat products in the dairy group.

### Calculation of target energy intake from each food group

To calculate the target energy intake from each food group, we began by calculating the serving size in grams of each food item on the short list according to DASH portion sizes, which differ for different items within a food group. Then, for each food item we calculated the energy content per serving. Next, we averaged the energy content per serving for all foods within a food group, and then multiplied this value by the total number of servings recommended in the DASH pattern to yield the target energy per food group (Table 1). Average serving sizes were extracted from DASH guidance documents^(^ ^53^ ^)^ and converted into grams using information from the US Department of Agriculture’s nutrient database^(^ ^54^ ^)^. Energy values were obtained from the West African Food Composition Tables^(^ ^55^ ^)^ where available; remaining values not present in the West African Food Composition Table were obtained from the US Department of Agriculture’s database^(^ ^54^ ^)^.

The target number of servings per food group per day was based on the DASH dietary pattern for a 2000 kcal (8368 kJ) diet^(^ ^53^ ^)^, although these are presented as ranges for many foods and thus require some interpretation. Specifically, for food groups for which a range was presented, the average value was used. We used values of 5·0 servings/d for meat (‘6 or less servings per day’) and 0·7 servings/d for nuts, seeds and legumes (‘4–5 servings per week’). The small serving size for meat should be noted: DASH specifies 1 oz (28·35 g) servings. This number of servings per day yielded a total energy content of 2079 kcal (8698 kJ)/d for the first scenario (classifying starchy fruits and vegetables with other starchy staples) and 2280 kcal (9540 kJ)/d for the second scenario (classifying starchy fruits and vegetables with other fruits and vegetables). Rather than recommend a higher or lower energy intake than the estimated population requirement of 2100 kcal (8786 kJ)/capita per d (described above), and to facilitate comparison between the two scenarios for the reference diet, we adjusted the number of servings by multiplying the number of servings suggested in the DASH 2000 kcal (8368 kJ)/capita per d plan by the ratio of 2100 kcal (8786 kJ)/2079 kcal (8698 kJ) and 2100 kcal (8786 kJ)/2280 kcal (9540 kJ), respectively, to achieve a total energy content of 2100 kcal (8786 kJ)/capita per d for each version of the reference diet (Table 1).

### Calculation of per capita energy supply using national data

For each of the seven DASH food groups, the per capita availability in the national food supply was estimated based on 2011 country-level data for Cameroon from the FBS^(^ ^56^ ^)^. The FAO calculates the quantity (metric tons) of food available for human consumption as the total supply (total quantity produced in country plus total quantity imported) minus quantities exported, fed to livestock, used for seed, used for industrial purposes, and lost during storage and transportation^(^ ^57^ ^)^. Per capita food supply estimates (kg/person) are then calculated by dividing the quantity of food available for human consumption by the population size. Finally, the per capita quantity of food available for human consumption is converted to kcal/capita per d by multiplying the edible portion of the per capita weight by its energy value indicated in food composition tables.

To calculate the per capita daily energy supply, we first assigned the food commodities listed in the 2011 Cameroon FBS to the seven DASH food groups. Next, we summed the energy supply of each food commodity as reported in the FBS (kcal/capita per d) for each of the seven food groups and for the total of all seven groups. As for the reference diet, we constructed separate scenarios with starchy fruits and vegetables categorized along with grains, or with their respective fruit and vegetable groups. Food commodities reported in the FBS that were not included in the DASH pattern (e.g. sugar, alcoholic beverages, coffee and spices) were excluded.

### Comparison of the national food supply with the reference diet

The energy supply (kcal/capita per d) for each food group was then compared with our calculations of target energy intake for the two scenarios described above. For each food group, the dietary gap was calculated as the energy available in the food supply minus the target energy intake.

## Results

**Fig. 1:**
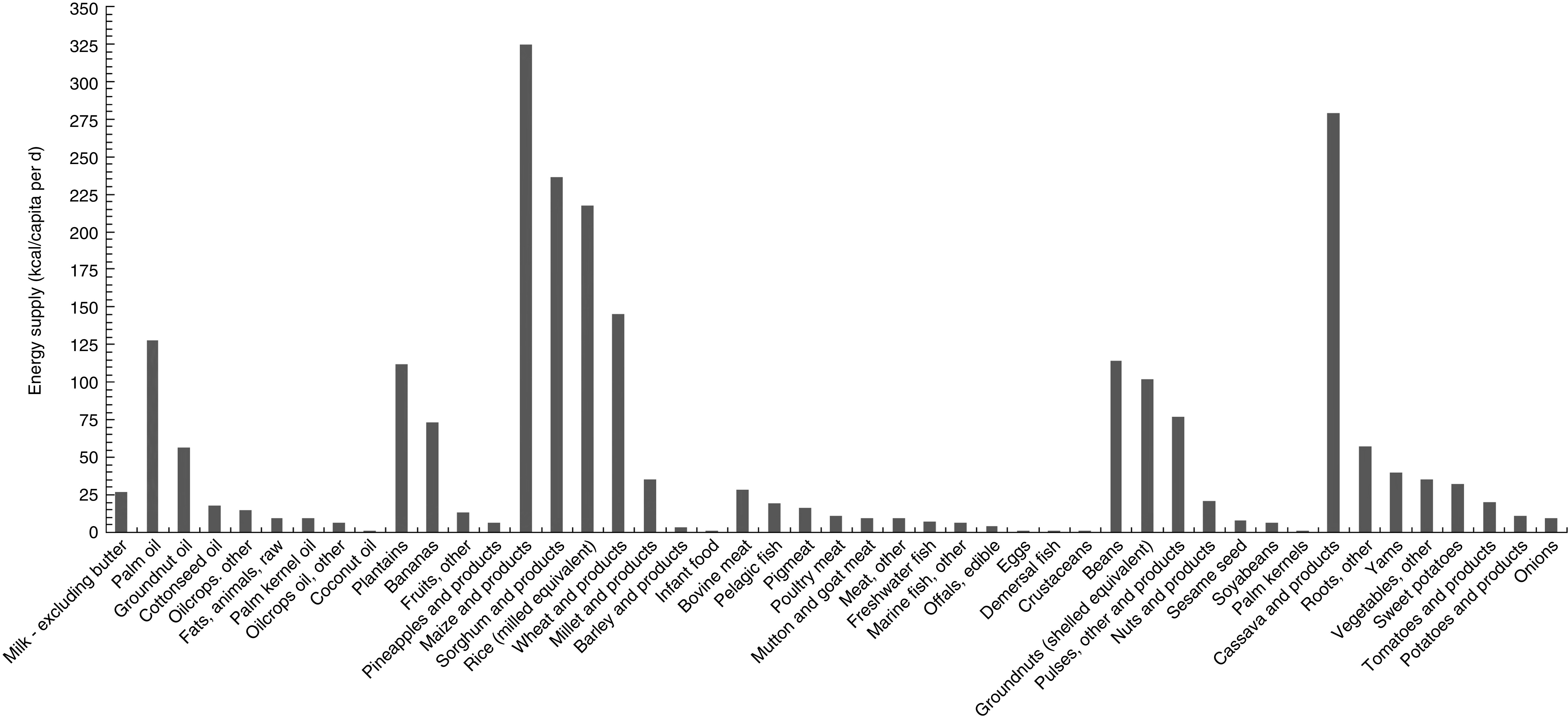
Per capita daily energy supply of foods in the 2011 Cameroon food supply (1 kcal=4·184 kJ)

The per capita daily energy supply of foods in the Cameroon 2011 food supply is depicted in Fig. 1. The foods that provided the largest amounts of energy were maize and cassava, providing 324 and 279 kcal (1356 and 1167 kJ)/capita per d, respectively. Other grains such as sorghum, rice and wheat were also major sources of energy. Among the fats and oils, palm oil was the predominant item (128 kcal (536 kJ)/capita per d). Among fruits, plantains provided more energy than all other fruits combined.

**Fig. 2:**
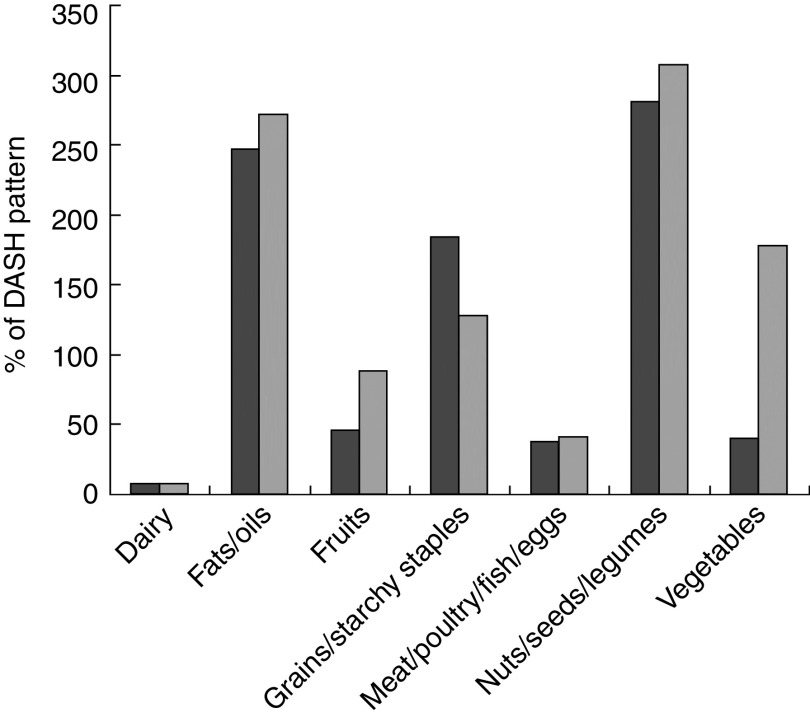
Gaps in the 2011 Cameroon food supply compared with the Dietary Approaches to Stop Hypertension (DASH) reference diet pattern: 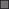, Scenario A; 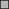, Scenario B. Gaps are expressed as a percentage of the DASH reference diet pattern provided by the food supply. Scenario A categorizes starchy fruit (plantains) and vegetables (potatoes, cassava, etc.) as starchy staples. Scenario B categorizes starchy fruit and vegetables in their respective fruit and vegetable groups

The Cameroon food supply is compared with the DASH reference diet for the two scenarios on a per capita daily energy basis in Table 2 and as a percentage of the DASH reference diet amounts in Fig. 2. For both scenarios, the largest deficit in the food supply was for dairy products: the deficit was 346–382 kcal (1148–1598 kJ)/capita per d and the supply provided only ~7 % of the energy from dairy recommended in the DASH diet. The second largest gap in the food supply was for meat/poultry/fish/eggs: the deficit was 161–188 kcal (674–787 kJ)/capita per d and the supply was 37–41 % of recommended in the DASH diet. In Scenario A (with starchy fruits and vegetables categorized in the starchy staples group) there were deficits of 111 and 96 kcal (464 and 402 kJ)/capita per d for fruits and vegetables, respectively, and an excess of 679 kcal (2841 kJ)/capita per d for starchy staples. By contrast, in Scenario B (with starchy fruits and vegetables categorized in their respective fruit and vegetable groups), the deficit for fruits was only 29 kcal (121 kJ)/capita per d and there was an excess of 211 kcal (883 kJ)/capita per d for ‘vegetables’; the excess of energy for starchy staples was reduced to 209 kcal (874 kJ)/capita per d. In both scenarios, the largest excesses in terms of percentage of the recommended DASH reference diet amounts were for the fats/oils and legumes/nuts/seeds groups, for which the supply was 250–300 % of the DASH reference diet amounts. Although there was an excess of energy available from fats and oils, 53 % came from palm oil. Other major sources of fats and oils included groundnut oil, cottonseed oil and animal fat. None of these provides a good source of *n*-3 fatty acids, which are important for good health^(^ ^58^ ^)^.

**Table 2:** Comparison of daily per capita energy of Cameroon food supply with the Dietary Approaches to Stop Hypertension (DASH) reference diet patterns derived for Cameroon

	Scenario A, starchy fruits and vegetables categorized with starchy staples		Scenario B, starchy fruits and vegetables categorized with fruits and vegetables	
Food group	Energy supply (kcal/capita per d)	Energy gap* (kcal/capita per d)	Energy supply (kcal/capita per d)	Energy gap* (kcal/capita per d)
Dairy	27	–382	27	–346
Fats and oils	242	144	242	153
Fruits	92	–111	204	–29
Grains (Starchy staples)	1492	679	961	209
Meat, poultry, fish and eggs	112	–188	112	–161
Nuts, seeds and legumes	329	212	329	222
Vegetables	64	–96	483	211
Total	2358	258	2358	258

1 kcal=4·184 kJ.

* Difference between the food supply and the Cameroon DASH reference diet pattern.

In addition to assessing differences in the dietary gap identified by our methods based on the way roots, tubers and plantains are classified (Scenarios A and B), we also explored the sensitivity of our methods to the type of dairy products that were selected to populate the dairy group. In particular, we substituted low-fat dairy products (1·5 % fat milk and low-fat yoghurt) in place of whole-fat dairy products in the DASH reference diet, given the limited availability and consumption of low-fat dairy products in Cameroon. If full-fat dairy foods were replaced with low-fat versions, the deficit in the dairy product supply in Cameroon would decrease by about 70 kcal (293 kJ)/capita per d, remaining very large (>200 kcal (>837 kJ)/capita per d). The gaps for the other food groups would change only slightly because the reduction in energy from dairy products in the low-fat dairy DASH reference diet pattern would be offset by slight increases in energy from all of the other food groups (to total to the target of 2100 kcal (8786 kJ)/capita per d).

## Discussion

We developed a methodology to assess the adequacy of a national food supply with respect to a healthy dietary pattern. Application of this method to a test country, Cameroon, revealed large gaps in availability of animal-source foods, with a deficit of ~350–380 kcal (~1464–1590 kJ)/capita per d for dairy products and another ~160–190 kcal (~669–795 kJ)/capita per d for meat, poultry, fish and eggs. When roots, tubers and plantains were categorized in the starchy staples group, there were deficits of ~100 kcal (~418 kJ)/capita per d for vegetables and ~110 kcal (~460 kJ)/capita per d for fruits, and an excess of ~680 kcal (~2845 kJ)/capita per d for starchy staples. When roots and tubers were categorized as vegetables and plantains were categorized as fruits, the deficit for ‘vegetables’ disappeared and the deficit for ‘fruits’ was reduced to ~30 kcal (~126 kJ)/capita per d. However, from a dietary perspective, classifying roots, tubers and plantains with grains is more appropriate because of the way these foods are typically consumed (as starchy staples rather than as side dishes) and their nutrient profile.

We selected the DASH diet as our reference diet because its efficacy for health promotion is based on evidence from a randomized intervention trial (albeit from a single country) and the recommendations are general enough to allow tailoring of specific foods within each food group to reflect local food preferences and supply. Similarly rigorous evidence for the potential health-promoting effects of major adaptations of the DASH diet (e.g. for a vegetarian version or one that is significantly more plant-based) is not yet available, making it challenging to identify an alternative to the DASH diet for broader use. It could be argued that some of the gaps identified for certain food groups may reflect the cultural context in which the DASH diet was developed (e.g. relatively high suggested intake of dairy products and lower consumption of nuts, seeds and legumes when compared with a diet more typical of West Africa), as discussed below. In general, however, our results reveal a food supply for Cameroon with, on average, adequate energy availability but shortfalls in the supply of foods that are micronutrient-dense. Because the DASH diet was developed with a focus on reduction in chronic disease risk, but micronutrient deficiencies are still prevalent in countries such as Cameroon, we conducted an exploratory analysis (see online supplementary material) to confirm whether the constructed healthy diet would improve adequacy of intake of five key micronutrients (vitamin A, vitamin B_12_, folate, Zn and Fe). We found that the hypothetical diet would greatly reduce the predicted prevalence of inadequate micronutrient intakes, compared with individual dietary intake data collected from women in a nationally representative dietary survey in Cameroon. Other analyses of FBS data suggest inadequate micronutrient density of the available food supply in Cameroon and elsewhere in sub-Saharan Africa, which is consistent with our observation of gaps in the supply of fruits, vegetables and animal-source foods^(^ ^38^ ^,^ ^39^ ^)^. Analyses of available Zn in the food supply conducted by Wessells and Brown^(^ ^39^ ^)^ and Arsenault *et al*.^(^ ^40^ ^)^ revealed a high prevalence of inadequate Zn availability (33 %) for Cameroon. FBS analyses by Arsenault *et al*. also predicted a high prevalence of inadequate vitamin A intakes in Cameroon, but not for folate and several other micronutrients^(^ ^40^ ^)^. However, these analyses assessed nutrient inadequacies rather than the overall dietary pattern.

There are several limitations associated with the methodology and data we used to estimate the dietary gap between a country’s food supply and the food that would be required for its population to achieve a ‘healthy’ diet. First, recommended food group consumption in the DASH diet and other food-based dietary guidelines is often expressed as a range of servings. We used the approximate midpoint of this range to operationalize the diet; future work could incorporate sensitivity analyses using both low and high values. Second, identifying a reference diet that is both widely culturally appropriate/adaptable as well as evidence-based is a challenge. Because the DASH diet was originally developed for application in the USA and thus reflects the dietary preferences of Americans, the recommended amounts for each food group (i.e. number of servings and/or serving sizes) may not be appropriate globally. For example, the DASH diet includes 85–170 g of lean meats, poultry and fish daily. When populated with foods commonly consumed in Cameroon and under the conservative assumptions that all dairy products are low-fat and all fats and oils are from plant sources, the DASH diet includes ~33 % of energy from animal-source foods. This is well above the estimated national availability of energy from animal-source foods in Cameroon, which is approximately 6 %^(^ ^56^ ^)^, and estimated intakes of animal-source foods among women and children (~7–9 % of total energy)^(^ ^59^ ^)^. In addition, research on the environmental and health impacts of intensive production and consumption of animal-source foods has sparked global dialogue on sustainable diets. It is possible that diets with a lower percentage of energy from animal-source foods may adequately promote health and be more affordable, culturally appropriate and sustainable in some settings. Another example is that the recommended consumption of nuts, seeds and legumes in the DASH diet is only three to five servings weekly. Given that there are health advantages to frequent consumption of legumes^(^ ^60^ ^)^ and nuts in particular^(^ ^61^ ^,^ ^62^ ^)^, a reference ‘healthy diet’ that incorporates at least one serving daily from this food group may be more appropriate. This may also make the reference ‘healthy diet’ a better fit for countries like Cameroon where legumes and seeds, such as groundnuts and squash or pumpkin seeds, are frequently consumed.

To address some of these issues, we made a rough comparison between the hypothetical DASH diet for Cameroon and the recently published Benin Food Guide, which aimed to contribute to national efforts to ‘halt the progression of chronic diseases while ensuring adequate intake to prevent deficiencies’^(^ ^63^ ^)^. The guide was developed using linear programming to identify optimal amounts of foods per food group that could meet both nutrient requirements and dietary guidelines, while being consistent with local food habits^(^ ^64^ ^)^. These amounts were then expressed as a daily number of servings, giving serving size examples. Given that the publicly available food guide includes only a few suggested foods, we were not able to completely translate the recommendations to perform a quantitative analysis as we did with the DASH diet. However, for a rough comparison we converted the recommended number of servings in each food group of the Benin Food Guide to the approximate amount of energy from each food group, and adjusted the resulting diet to total 2100 kcal (8786 kJ). In terms of dietary gaps, the two patterns result in similar supply shortfalls of fruits and vegetables. The Benin Food Guide places both plant- and animal-source protein-rich foods into a single food group and thus allows for meeting recommendations for that food group without consuming any animal-source foods; this means that the gap in meat, poultry, fish and eggs identified using DASH would be eliminated. There would still be a gap in dairy products using the Benin Food Guide, but it would be about half the size of the gap identified using the DASH diet. Thus, if using the Benin Food Guide, certain gaps would be less evident or smaller. However, it is not clear whether this approach would result in similar health benefits as following a DASH-type diet because no intervention trials have been conducted based on the Benin Food Guide (which is the case for all national food-based dietary guidelines to date). For example, it is likely that without requiring any animal-source foods in the ‘protein’ food group, intakes of certain micronutrients would be lower (e.g. Ca, Fe, Zn, vitamin B_12_), with potential implications for some health outcomes. Ideally, intervention studies for dietary patterns adapted for specific countries would be tested in settings for which they are intended. However, due to the cost and complexity of such studies, it will be necessary to take steps to address the growing burden of chronic diseases in low-income settings before such information is available.

Several limitations of the FBS data also merit note because of potential implications for the accuracy and application of our estimates^(^ ^65^ ^)^. First, the FAO estimates of the quantity of food available for human consumption may not fully or accurately capture non-commercial or subsistence production, which can be substantial in developing countries. Second, the estimates do not account for wastage at the household level (e.g. losses during household storage or food preparation, as feed for domestic animals/pets, etc.). Finally, the FBS data do not allow for analysis of seasonal or geographic differences in the food supply, nor do they account for variability in access and utilization by gender, age and socio-economic status subgroups^(^ ^4^ ^,^ ^66^ ^)^. Because the data are provided at the country level and at an annual time step, food distribution, access and utilization issues are not reflected in our analysis, and our estimates represent the annual average amount of energy available per person per day. Despite these limitations, the FBS data are of great utility because they are made publicly available on an annual basis for a very comprehensive set of countries and the methods used to estimate national-level food supplies are, to the extent possible, fairly consistent across countries^(^ ^39^ ^,^ ^40^ ^)^. While dietary gaps can be assessed using household- or individual-level food consumption data, which allow for disaggregation, these data are influenced by many factors such as preferences and economic access and do not directly represent national food availability.

The ‘dietary gap assessment’ approach described herein can be expanded to other countries using available information on commonly consumed foods to compile appropriate food groupings for a country-specific DASH pattern. We used Cameroon as a case study because we had access to recent individual-level nationally representative dietary intake information in order to populate the DASH food groups with country-specific foods, but this type of information is not available for most countries. Another potential source of information on commonly consumed foods are nationally representative household consumption and expenditure surveys which are conducted in many developing countries by government statistical services or through the World Bank’s Living Standards Measurement Study, usually more widely and frequently than individual-level consumption studies. Household consumption and expenditure surveys typically include a list of foods known to be consumed in the country and respondents are asked how much of each food the household acquired and/or consumed during some reference period. Other options include more disaggregated data available from FAOSTAT on production and trade amounts of foods for each country, although these data are somewhat limited in completeness, or published reports from local dietary intake assessments.

## Conclusions and implications

Many countries are likely to have large gaps in supply of animal-source foods, fruits and vegetables relative to the amounts needed to achieve ‘healthy diet’ patterns. Some of these gaps are evident even in high-income countries. For example, in the USA, analyses of the food supply conducted over the course of several decades have repeatedly demonstrated inadequate supply of fruits and vegetables to meet US Government dietary recommendations^(^ ^25^ ^,^ ^67^ ^,^ ^68^ ^)^. Similarly, Siegel *et al*. documented inadequate supply of fruits and vegetables at the global level^(^ ^37^ ^)^.

Concern regarding the ability of the food system to nourish a growing population has precipitated numerous scientific and high-level meetings^(^ ^69^ ^)^. Although there needs to be continued attention to the overall adequacy of the global food supply to meet energy needs, a focus on staple food production will not address the need for greater dietary diversity and healthier dietary patterns^(^ ^70^ ^)^.

The type of analysis presented in the current paper highlights the need for and can help inform the development of strategies to increase both the supply and demand for the foods needed for a healthy diet. Action is needed on several fronts, including increased production, increased demand and reduced post-harvest losses and other wastage, accompanied by broader strategies aimed at reducing inequity in access to affordable healthy foods. Country-level dietary gap analysis, as illustrated herein, could provide a useful contribution to ongoing policy dialogues on agricultural and food system priorities. This information may be particularly useful in discussions related to policies and investments that promote improved production of and access to more diverse foods^(^ ^23^ ^)^, and would complement existing economic and ecological analyses that support the benefit of greater diversification^(^ ^71^ ^,^ ^72^ ^)^.

## Supplementary material

For supplementary material accompanying this paper visit https://doi.org/10.1017/S1368980017001173. click here to view supplementary material

## References

[1] Food and Agriculture Organization of the United Nations & World Health Organization (2014) Rome Declaration on Nutrition. Second International Conference on Nutrition, Rome, 19–21 November 2014. http://www.fao.org/3/a-ml542e.pdf (accessed May 2015).

[2] Food and Agriculture Organization of the United Nations (1996) Rome Declaration on World Food Security. World Food Summit, 13–17 November 1996, Rome, Italy. http://www.fao.org/docrep/003/w3613e/w3613e00.htm (accessed May 2015).

[3] Herforth A & Ahmed S (2015) The food environment, its effects on dietary consumption, and potential for measurement within agricultural-nutrition interventions. Food Secur 7, 505–520.

[4] Food and Agriculture Organization of the United Nations (2016) Supply Utilization Accounts and Food Balance Sheets: background information for your better understanding. http://www.fao.org/economic/the-statistics-division-ess/methodology/methodology-systems/supply-utilization-accounts-and-food-balance-sheets-background-information-for-your-better-understanding/en/ (accessed September 2016).

[5] Vogt TM, Appel LJ, Obarzanek E et al. (1999) Dietary Approaches to Stop Hypertension: rationale, design, and methods. DASH Collaborative Research Group. J Am Diet Assoc 99, Suppl. 8, S12–S18. 10450289 10.1016/s0002-8223(99)00411-3

[6] Struijk EA, May AM, Wezenbeek NL et al. (2014) Adherence to dietary guidelines and cardiovascular disease risk in the EPIC-NL cohort. Int J Cardiol 176, 354–359. 25107447 10.1016/j.ijcard.2014.07.017

[7] Jankovic N, Geelen A, Streppel MT et al. (2015) WHO guidelines for a healthy diet and mortality from cardiovascular disease in European and American elderly: the CHANCES project. Am J Clin Nutr 102, 745–756. 26354545 10.3945/ajcn.114.095117 PMC4588736

[8] Arimond M, Wiesmann D, Becquey E et al. (2010) Simple food group diversity indicators predict micronutrient adequacy of women’s diets in 5 diverse, resource-poor settings. J Nutr 140, issue 11, 2059S–2069S. 20881077 10.3945/jn.110.123414 PMC2955880

[9] Food and Agriculture Organization of the United Nations, International Fund for Agricultural Development & World Food Programme (2013) The State of Food Insecurity in the World 2013: The Multiple Dimensions of Food Security. Rome: FAO; available at http://www.fao.org/docrep/018/i3434e/i3434e.pdf

[10] Popkin BM, Richards MK & Montiero CA (1996) Stunting is associated with overweight in children of four nations that are undergoing the nutrition transition. J Nutr 126, 3009–3016. 9001368 10.1093/jn/126.12.3009

[11] Popkin BM & Gordon-Larsen P (2004) The nutrition transition: worldwide obesity dynamics and their determinants. Int J Obes Relat Metab Disord 28, Suppl. 3, S2–S9. 15543214 10.1038/sj.ijo.0802804

[12] Gomez MI, Barrett CB, Raney T et al. (2013) Post-green revolution food systems and the triple burden of malnutrition. Food Policy 42, 129–138.

[13] Vorster HH, Kruger A & Margetts BM (2011) The nutrition transition in Africa: can it be steered into a more positive direction? Nutrients 3, 429–441. 22254104 10.3390/nu3040429 PMC3257689

[14] Green R, Sutherland J, Dangour AD et al. (2016) Global dietary quality, undernutrition and non-communicable disease: a longitudinal modelling study. BMJ Open 6, e009331. 10.1136/bmjopen-2015-009331 PMC4716260 26758259

[15] Roberto CA, Swinburn B, Hawkes C et al. (2015) Patchy progress on obesity prevention: emerging examples, entrenched barriers, and new thinking. Lancet 385, 2400–2409. 25703111 10.1016/S0140-6736(14)61744-X

[16] Ng M, Fleming T, Robinson M et al. (2013) Global, regional, and national prevalence of overweight and obesity in children and adults during 1980–2013: a systematic analysis for the Global Burden of Disease Study. Lancet 384, 766–781. 10.1016/S0140-6736(14)60460-8 PMC4624264 24880830

[17] NCD Risk Factor Collaboration (2016) Trends in adult body-mass index in 200 countries from 1975 to 2014: a pooled analysis of 1698 population-based measurement studies with 19.2 million participants. Lancet 387, 1377–1396. 27115820 10.1016/S0140-6736(16)30054-X PMC7615134

[18] NCD Risk Factor Collaboration (2016) Worldwide trends in diabetes since 1980: a pooled analysis of 751 population-based studies with 4.4 million participants. Lancet 387, 1513–1530. 27061677 10.1016/S0140-6736(16)00618-8 PMC5081106

[19] Jaacks LM, Slining MM & Popkin BM (2015) Recent underweight and overweight trends by rural–urban residence among women in low- and middle-income countries. J Nutr 145, 352–357. 25644358 10.3945/jn.114.203562 PMC6619682

[20] Suchdev PS, Shah A, Jefferds ME et al. (2013) Sustainability of market-based community distribution of Sprinkles in western Kenya. Matern Child Nutr 9, Suppl. 1, S78–S88. 10.1111/j.1740-8709.2012.00450.x PMC6860854 23167586

[21] Darnton-Hill I & Nalubola R (2002) Fortification strategies to meet micronutrient needs: successes and failures. Proc Nutr Soc 61, 231–241. 12133205 10.1079/PNS2002150

[22] Johns T, Powell B, Maundu P et al. (2013) Agricultural biodiversity as a link between traditional food systems and contemporary development, social integrity and ecological health. J Sci Food Agric 93, Suppl. 1, S3433–S3442. 10.1002/jsfa.6351 23963831

[23] Johnston JL, Fanzo JC & Cogill B (2014) Understanding sustainable diets: a descriptive analysis of the determinants and processes that influence diets and their impact on health, food security, and environmental sustainability. Adv Nutr 5, 418–429. 25022991 10.3945/an.113.005553 PMC4085190

[24] Hawkes C (2006) Uneven dietary development: linking the policies and processes of globalization with the nutrition transition, obesity and diet-related chronic diseases. Global Health 2, 4. 16569239 10.1186/1744-8603-2-4 PMC1440852

[25] Kantor L (1999) A comparison of the US food supply with the Food Guide Pyramid recommendations. In *America’s Eating Habits: Changes and Consequences*. *Agriculture Information Bulletin* , no. AIB-750, pp. 71–95 [E Frazão, editor]. Washington: DC: US Department of Agriculture, Economic Research Service; available at https://www.ers.usda.gov/webdocs/publications/42215/5833_aib750d_1_.pdf?v=41055

[26] Food and Agriculture Organization of the United Nations (2016) Food-based dietary guidelines. http://www.fao.org/nutrition/education/food-dietary-guidelines/home/en/ (accessed July 2016).

[27] Food and Agriculture Organization of the United Nations & World Health Organization (1998) *Preparation and Use of Food-Based Dietary Guidelines. Report of a Joint FAO/WHO Consultation. WHO Technical Report Series* no. 880. Geneva: WHO; available at http://whqlibdoc.who.int/trs/WHO_TRS_880.pdf

[28] Dwyer JT (2012) Dietary standards and guidelines: similarities and differences among countries. In Present Knowledge in Nutrition, 10th ed., pp. 1110–1134 [JW Erdman, editor]. Hoboken, NJ: Wiley-Blackwell.

[29] Sofi F, Macchi C, Abbate R et al. (2014) Mediterranean diet and health status: an updated meta-analysis and a proposal for a literature-based adherence score. Public Health Nutr 17, 2769–2782. 24476641 10.1017/S1368980013003169 PMC10282340

[30] Mithril C, Dragsted LO, Meyer C et al. (2012) Guidelines for the New Nordic Diet. Public Health Nutr 15, 1941–1947. 22251407 10.1017/S136898001100351X

[31] SacksFM, AppelLJ, MooreTJ et al. (1999) A dietary approach to prevent hypertension: a review of the Dietary Approaches to Stop Hypertension (DASH) Study. Clin Cardiol 22, Suppl. 3, S6–S10.10.1002/clc.496022150310410299

[32] Harmon BE, Boushey CJ, Shvetsov YB et al. (2015) Associations of key diet-quality indexes with mortality in the Multiethnic Cohort: the Dietary Patterns Methods Project. Am J Clin Nutr 101, 587–597. 25733644 10.3945/ajcn.114.090688 PMC4340063

[33] Mozaffarian D (2016) Dietary and policy priorities for cardiovascular disease, diabetes, and obesity: a comprehensive review. Circulation 133, 187–225. 26746178 10.1161/CIRCULATIONAHA.115.018585 PMC4814348

[34] World Health Organization (2015) Healthy diet. Fact sheet no. 394. http://www.who.int/mediacentre/factsheets/fs394/en/ (accessed July 2016).

[35] Siervo M, Lara J, Chowdhury S, Ashor A et al. (2014) Effects of the Dietary Approach to Stop Hypertension (DASH) diet on cardiovascular risk factors: a systematic review and meta-analysis. Br J Nutr 113, 1–15. 25430608 10.1017/S0007114514003341

[36] Phillips KM, Stewart KK, Karanja NM et al. (1999) Validation of diet composition for the Dietary Approaches to Stop Hypertension trial. DASH Collaborative Research Group. J Am Diet Assoc 99, Suppl. 8, S60–S68. 10450296 10.1016/s0002-8223(99)00418-6

[37] Siegel KR, Ali MK, Srinivasiah A et al. (2014) Do we produce enough fruits and vegetables to meet global health need? PLoS One 9, e104059. 25099121 10.1371/journal.pone.0104059 PMC4123909

[38] Joy EJ, Ander EL, Black CR et al. (2014) Dietary mineral supplies in Africa. Physiol Plant 151, 208–229. 24524331 10.1111/ppl.12144 PMC4235459

[39] Wessells KR & Brown KH (2012) Estimating the global prevalence of zinc deficiency: results based on zinc availability in national food supplies and the prevalence of stunting. PLoS One 7, e50568. 23209782 10.1371/journal.pone.0050568 PMC3510072

[40] Arsenault JE, Brown KH & Hijmans RJ (2015) Improving nutrition security through agriculture: an analytical framework based on national food balance sheets to estimate nutrition adequacy of food supplies. Food Secur 7, 693–707.

[41] Harsha DW, Lin PH, Obarzanek E et al. (1999) Dietary Approaches to Stop Hypertension: a summary of study results. DASH Collaborative Research Group. J Am Diet Assoc 99, Suppl. 8, S35–S39. 10450292 10.1016/s0002-8223(99)00414-9

[42] World Bank (2015) Urban population (% of total). http://data.worldbank.org/indicator/SP.URB.TOTL.IN.ZS?view=chart (accessed September 2016).

[43] International Food Policy Research Institute (2015) Global Nutrition Report 2015: Actions and Accountability to Advance Nutrition and Sustainable Development. Washington, DC: IFPRI; available at 10.2499/9780896298835 PMC4424785 25979494

[44] Engle-Stone R, Ndjebayi AO, Nankap M et al. (2015) Prevalence of obesity and elevated waist-to-hip ratio among a national sample of women of reproductive age in Cameroon, and change from 2009 to 2012 in urban areas. Poster presented at *Experimental Biology 2015*, Boston, MA, USA, 28 March–1 April 2015.

[45] United Nations High Commissioner for Refugees, UNICEF, World Food Programme *et al*. (2004) Food and Nutrition Needs in Emergencies. Geneva: WHO; available at http://www.who.int/nutrition/publications/emergencies/a83743/en/

[46] Republic of Cameroon (2010) La population du Cameroun en 2010. http://www.statistics-cameroon.org/downloads/La_population_du_Cameroun_2010.pdf (accessed August 2014).

[47] Engle-Stone R, Ndjebayi AO, Nankap M et al. (2012) Consumption of potentially fortifiable foods by women and young children varies by ecological zone and socio-economic status in Cameroon. J Nutr 142, 555–565. 22323765 10.3945/jn.111.148783

[48] Agudo A (2005) Measuring Intake of Fruit and Vegetables. Geneva: WHO; available at http://www.who.int/dietphysicalactivity/publications/f&v_intake_measurement.pdf

[49] World Cancer Research Fund & American Institute for Cancer Research (2007) Food, Nutrition, Physical Activity, and the Prevention of Cancer: A Global Perspective. Washington, DC: AICR; available at http://www.aicr.org/assets/docs/pdf/reports/Second_Expert_Report.pdf

[50] World Health Organization (2010) Indicators for Assessing Infant and Young Child Feeding Practices. Part II: Measurement. Geneva: WHO; available at http://www.who.int/nutrition/publications/infantfeeding/9789241599290/en/

[51] Food and Agriculture Organization of the United Nations (2011) Guidelines for measuring household and individual dietary diversity. http://www.fao.org/fileadmin/user_upload/wa_workshop/docs/FAO-guidelines-dietary-diversity2011.pdf (accessed July 2014).

[52] Karanja NM, Obarzanek E, Lin PH et al. (1999) Descriptive characteristics of the dietary patterns used in the Dietary Approaches to Stop Hypertension Trial. DASH Collaborative Research Group. J Am Diet Assoc 99, Suppl. 8, S19–S27. 10450290 10.1016/s0002-8223(99)00412-5

[53] US Department of Health and Human Services, National Heart, Lung, and Blood Institute (2006) In Brief: Your guide to lowering your blood pressure with DASH. https://www.nhlbi.nih.gov/files/docs/public/heart/dash_brief.pdf (accessed June 2014).

[54] US Department of Agriculture (2011) National Nutrient Database for Standard Reference, Release 27. http://ndb.nal.usda.gov/ (accessed August 2014).

[55] Food and Agriculture Organization of the United Nations (2012) West African Food Composition Table. Rome: FAO; available at http://www.fao.org/docrep/015/i2698b/i2698b00.pdf

[56] Food and Agriculture Organization of the United Nations (2011) Food Balance Sheets. http://www.fao.org/faostat/en/#data/FBS (accessed July 2014).

[57] Food and Agriculture Organization of the United Nations (2001) Food Balance Sheets: a handbook. http://www.fao.org/docrep/003/x9892e/x9892e00.htm (accessed July 2014).

[58] Calder PC & Yaqoob P (2009) Omega-3 polyunsaturated fatty acids and human health outcomes. Biofactors 35, 266–272. 19391122 10.1002/biof.42

[59] Shahab-Ferdows S, Engle-Stone R, Hampel D et al. (2015) Regional, socioeconomic, and dietary risk factors for vitamin B-12 deficiency differ from those for folate deficiency in Cameroonian women and children. J Nutr 145, 2587–2595. 26446486 10.3945/jn.115.210195

[60] Rebello CJ, Greenway FL & Finley JW (2014) A review of the nutritional value of legumes and their effects on obesity and its related co-morbidities. Obes Rev 15, 392–407. 24433379 10.1111/obr.12144

[61] Jackson CL & Hu FB (2014) Long-term associations of nut consumption with body weight and obesity. Am J Clin Nutr 100, Suppl. 1, S408–S411. 10.3945/ajcn.113.071332 PMC4144111 24898229

[62] Grosso G, Yang J, Marventano S et al. (2015) Nut consumption on all-cause, cardiovascular, and cancer mortality risk: a systematic review and meta-analysis of epidemiologic studies. Am J Clin Nutr 101, 783–793. 25833976 10.3945/ajcn.114.099515

[63] Republic of Benin (2015) Benin Food Guide. http://poledfn.org/wp-content/uploads/2014/03/guide_alimentaire_benin_legal.pdf (accessed November 2016).

[64] Levesque S, Delisle H & Agueh V (2015) Contribution to the development of a food guide in Benin: linear programming for the optimization of local diets. Public Health Nutr 18, 622–631. 24762926 10.1017/S1368980014000706 PMC10271684

[65] Del Gobbo LC, Khatibzadeh S, Imamura F et al. (2015) Assessing global dietary habits: a comparison of national estimates from the FAO and the Global Dietary Database. Am J Clin Nutr 101, 1038–1046. 25788002 10.3945/ajcn.114.087403 PMC4409685

[66] Jones AD, Ngure FM, Pelto G et al. (2013) What are we assessing when we measure food security? A compendium and review of current metrics. Adv Nutr 4, 481–505. 24038241 10.3945/an.113.004119 PMC3771133

[67] Miller PE, Reedy J, Kirkpatrick SI et al. (2015) The United States food supply is not consistent with dietary guidance: evidence from an evaluation using the Healthy Eating Index-2010. J Acad Nutr Diet 115, 95–100. 25441965 10.1016/j.jand.2014.08.030 PMC4276446

[68] Krebs-Smith SM, Reedy J & Bosire C (2010) Healthfulness of the US food supply: little improvement despite decades of dietary guidance. Am J Prev Med 38, 472–477. 20153133 10.1016/j.amepre.2010.01.016 PMC2858769

[69] Herforth A, Lidder P & Gill M (2015) Strengthening the links between nutrition and health outcomes and agricultural research. Food Secur 7, 457–461.

[70] Pingali P (2015) Agricultural policy and nutrition outcomes – getting beyond the preoccupation with staple grains. Food Secur 7, 583–591.

[71] Kuteya A & Sitko NJ (2015) Creating scarcity from abundance: bumper harvests, high prices, and the role of state interventions in Zambian maize markets. Afr J Agric Nutr Dev 15, 10272–10289.

[72] Remans R, Flyn DF & DeClerck F (2011) Assessing nutritional diversity of cropping systems in African villages. PLoS One 6, e21235. 21698127 10.1371/journal.pone.0021235 PMC3116903

