# Supplementary material for: Dietary gap assessment: an approach for evaluating whether a country’s food supply can support healthy diets at the population level
Source: Public Health Nutr. 2017 Jun 21;20(13):2277–88. doi: 10.1017/S1368980017001173 (PMC5582405; doi:10.1017/S1368980017001173)
Supplement: Supplementary file 1 [file S1368980017001173sup001.docx]

**Online Supplementary Material**

**Dietary gap assessment: an approach for evaluating whether a country’s food supply can support healthy diets at the population level**

***Comparing adequacy of intake of selected micronutrients from the reference diet and from actual diets***

***Methods***

As an exploratory analysis, we examined the extent to which the DASH diet might meet micronutrient needs, and/or increase micronutrient intakes relative to current intakes in the Cameroon context. As an example, we chose to focus on consumption of five micronutrients (vitamin A, vitamin B-12, folate, zinc and iron) by women, because low intakes and status of each nutrient have been documented among women in Cameroon (although the prevalence varies widely by region).^(1-3)^ To estimate the micronutrient adequacy of the DASH diet in the Cameroon context, we first calculated the total amount of each micronutrient provided by each food group and by the whole diet. Information on micronutrient content was obtained from the West African Food Composition Tables^(4)^ where available, or from the USDA database^(5)^. The average micronutrient content of each food group from the Cameroon DASH diet was calculated as a weighted average of the micronutrient content of each of the “short list” foods in each food group. The contribution of each food was weighted by the energy (kcal/capita/day) provided by that food in the FBS data. We assumed that the coefficient of variation (CV) of the usual intake distribution was 45% for vitamin A, 30% for folate, 25% for zinc^(6)^ and 90% for vitamin B-12. We also assumed that the distribution of intakes was lognormal for vitamins A and B-12 and normal for folate and zinc. We then calculated the prevalence of predicted inadequate intakes (proportion of intakes below the estimated average requirement (EAR)) by applying the EAR cut-point method to a distribution with the specified mean and CV. EAR values from the US Institute of Medicine were used, with the exception of zinc, for which we used the IZiNCG EAR for unrefined, cereal-based diets. Because assessment of adequacy of iron intake requires use of the probability method, we estimated total iron intake but not prevalence of inadequate intakes. To compare the estimated micronutrient adequacy of the Cameroon DASH diet with actual dietary intakes measured among women in the national survey, we repeated the process above using published values for the women’s mean vitamin intakes, ^(2, 3)^ after adjustment of these values to represent intake at 2100 kcal/capita/day (assuming equal nutrient density). For example, mean intake of 4.50 µg/d vitamin B12 at 2180 kcal/ capita/day was adjusted to mean intake of 4.33 µg B12/d at 2100 kcal/capita/day.

***Results***

Mean micronutrient intakes among women in the dietary survey, adjusted to reflect a 2100 kcal diet, were 442 µg RAE/d for vitamin A, 4.33 µg B-12/d for vitamin B12, 289 µg DFE/d for folate, 8.2 mg/d for zinc, and 11.2 mg/d for iron. ^(2, 3)^ The predicted prevalence of intakes below the estimated average requirement was 69% for vitamin A, 27% for vitamin B-12, 64% for folate, and 28% for zinc. By contrast, for the Cameroon-equivalent of the DASH diet, the estimated mean nutrient intakes would be 846 µg RAE/d for vitamin A, 5.96 µg B-12/d, 384 µg DFE/d for folate, 12.4 mg/d for zinc, and 16.0 mg/d for iron. At these values, the predicted percentage with inadequate intakes would be reduced to 16% for Vitamin A, 15% for vitamin B-12, 29% for folate, and 4% for zinc.  It should be noted that these calculations do not account for reduced fractional absorption of vitamin B-12 from servings of food with high B-12 content, which would increase the observed prevalence of inadequate B-12 intakes.^(7)^ While exploratory, these results suggest that a dietary pattern similar to the DASH diet (with increased consumption of fruits, vegetables, and animal-source foods) could contribute to increasing micronutrient intakes.

***References***

1. Bosu WK (2015) An overview of the nutrition transition in West Africa: implications for non-communicable diseases. *Proc Nutr Soc* **74,** 466-477. DOI: 10.1017/s0029665114001669

2. Engle-Stone R, Ndjebayi AO, Nankap M, *et al.* (2014) Stunting prevalence, plasma zinc concentrations, and dietary zinc intakes in a nationally representative sample suggest a high risk of zinc deficiency among women and young children in Cameroon. *J Nutr* **144**, 382-91. DOI: 10.3945/jn.113.188383.

3. Engle-Stone R, Nankap M, Ndjebayi AO & Brown KH (2014) Simulations based on representative 24-h recall data predict region-specific differences in adequacy of vitamin A intake among Cameroonian women and young children following large-scale fortification of vegetable oil and other potential food vehicles. *J Nutr* **144,** 1826-1834. DOI: 10.3945/jn.114.195354

4. FAO (2012) West African Food Composition Table. Rome, Italy. <http://www.fao.org/docrep/015/i2698b/i2698b00.pdf>. Accessed December 24, 2015.

5. US Department of Agriculture (2011) National Nutrient Database for Standard Reference. Release 27. <http://ndb.nal.usda.gov/>. Accessed: August 8, 2014.

6. Wessells KR & Brown KH (2012) Estimating the global prevalence of zinc deficiency: results based on zinc availability in national food supplies and the prevalence of stunting. *PloS One* **7**:e50568.

7. Shahab-Ferdows S, Engle-Stone R, Hampel D, *et al.* (2015) Regional, Socioeconomic, and Dietary Risk Factors for Vitamin B-12 Deficiency Differ from Those for Folate Deficiency in Cameroonian Women and Children. *J Nutr* **145,** 2587-2595. DOI: 10.3945/jn.115.210195
